# Limitations and Potential Improvement of Cognitive Screening Tests for Mild Cognitive Impairment

**DOI:** 10.1002/alz70857_107231

**Published:** 2025-12-26

**Authors:** Dylan Hruskar, Daniel Z. Press

**Affiliations:** ^1^ Beth Israel Deaconess Medical Center, Boston, MA, USA

## Abstract

**Background:**

Mild cognitive impairment (MCI), an early sign of Alzheimer's Disease (AD), involves disruptions in both memory and cognition. Early and accurate detection of MCI is critical, as recently FDA approved disease modifying treatments are most effective at this stage of AD. The most commonly used screening tests for MCI are the Montreal Cognitive Assessment (MoCA) and the Mini‐Mental Status Examination (MMSE). However, these tests are time consuming, and item level analysis has not been conducted to see whether the components of these tests are sufficiently accurate in detecting MCI. This study aims to analyze the accuracy and actual contribution of individual items on the MoCA and the MMSE in MCI detection.

**Methods:**

Using the National Alzheimer's Coordinating Center's (NACC) data repository, our team conducted item level analysis of the MoCA and MMSE given to MCI as well as cognitively normal (CN) participants. We used area under the receiver operating characteristic curves (AUC‐ROC) to analyze the accuracy of these tests. This method enabled us to determine the overall accuracy in terms of MCI detection of the tests as well as the individual contribution of each item on the test.

**Results:**

The overall MoCA AUC‐ROC was 0.81 while the MMSE AUC‐ROC was 0.76, which shows that both tests are moderately accurate. Item level analysis of the tests revealed that only a small subset of components effectively contributes to MCI detection. We found that a six‐item combination of memory recall and orientation testing components contributes to over 98% of full test accuracy.

**Conclusion:**

In this study, we found that the MoCA and the MMSE have moderate efficacy in detecting MCI, and that only a subset of items from each test are the large contributors of MCI detection. Our analysis shows that the number of items on these tests could be substantially reduced which would lower time of test administration without loss of MCI detection effectiveness. There are opportunities for MCI screening test optimization, and further investigation is needed to determine the best possible combination of test items that would yield the highest detection accuracy in the lowest amount of time.

